# Estrogen, mitochondria, and growth of cancer and non-cancer cells

**DOI:** 10.1186/1477-3163-4-1

**Published:** 2005-01-15

**Authors:** Quentin Felty, Deodutta Roy

**Affiliations:** 1 Department of Environmental Health Sciences, University of Alabama at Birmingham, Birmingham, AL, 35294-0022 USA

## Abstract

In this review, we discuss estrogen actions on mitochondrial function and the possible implications on cell growth. Mitochondria are important targets of estrogen action. Therefore, an in-depth analysis of interaction between estrogen and mitochondria; and mitochondrial signaling to nucleus are pertinent to the development of new therapy strategies for the treatment of estrogen-dependent diseases related to mitochondrial disorders, including cancer.

## Introduction

Estrogen is considered to elicit different growth responses in various tissues through binding to the estrogen receptor (ER) α and ERβ [1-3]. The modulation of estrogen responsive gene transcription by the ER is termed a "*genomic*" action of estrogen. In contrast, "*non-genomic*" effects of estrogen are characterized by a rapid onset of action within seconds to minutes after hormone exposure through post-translation modification of signaling proteins. ER-mediated signaling pathways are considered to support the growth of normal, preneoplastic and neoplastic cells [1-3]. In contrast to the classical genomic pathways of estrogen action that occur over the course of several hours or days, recent studies have shown evidence of a rapid signaling pathway mediated by cell surface ERs and non-genomic estrogen-induced signal transduction pathways which contribute to cell proliferation [4]. Recently, we have identified that estrogen stimulated the growth of HEK 293 cells in an ER-independent manner given that this cell line does not contain ER (unpublished Singh KP, Venkat S, Roy D). These findings suggest that in addition to ER mediated actions, other factor(s) must be involved in the stimulation of cell growth by estrogen. More recently, mitochondria have been implicated in the control of cell proliferation [5]. For instance, the mitochondrial peripheral benzodiazepine receptor (PBR) has been implicated in the regulation of human breast cancer cell proliferation [6]. Similarly, we have demonstrated that mitochondria can modulate the expression of nuclear cell cycle genes and human breast tumor growth [7,8]. For instance, the growth of estrogen-dependent and estrogen-independent cells, is inhibited by controlling mitochondrial biogenesis [8]. In this paper, we critically review the role of mitochondria in the growth of estrogen-dependent cancer and non-cancer cells. Mitochondria are important targets of estrogen action. The cross-talk between the cell nucleus and the mitochondria appears to control estrogen-induced signaling involved in the apoptosis, proliferation, and differentiation of both normal and malignant cells. Mitochondria through its interaction with the cytoskeleton, export of cleaved signaling peptides, or generation of ROS appears to transduce signals to the nucleus for the activation of transcription factors involved in the cell cycle progression of estrogen-dependent cells. The understanding of the regulation of mitochondrial biogenesis by estrogenic compounds would open a new way to better understand steroidal and non-steroidal estrogen action at the cellular level.

### Estrogen actions at the mitochondria

Besides apoptosis, respiration, and oxidative phosphorylation; mitochondria also control ion homeostasis and the synthesis of heme, lipids, amino acids, and nucleotides. Steroidogenesis is also controlled by mitochondria. Estrogen biosynthesis-related enzymes, 3 β-hydroxysteroid dehydrogenase and aromatase, have been demonstrated in the mitochondria of ovarian tumor epithelial cells [9].

#### Estrogen transport to mitochondria

Although estrogen synthesis occurs in the mitochondria, exogenously added estrogen is also transported to this organelle. For instance, *in vivo *exposure of ovariectomized rats to tritiated 17-β-estradiol (E2) showed with increasing time, the translocation of this hormone from the plasmalemma mainly to the mitochondria (75%) rather than the nuclei in liver, adrenal gland, and spleen tissues [10]. The lipophilic property of E2 allows this molecule to easily diffuse into lipid bilayers. Since mitochondria are enriched with lipids, the organelle has the ability to act as an estrogen-sink within the cell. Although passive diffusion of estrogen into the mitochondria exists, a rapid delivery of E2 via receptor-mediated endocytosis from the plasma membrane to the mitochondria has been reported as a potential new pathway in HepG2 cells [11]. The uptake of E2-BSA from the medium by HepG2 cells occurred as early as 30 min. post-exposure and the ligand could be viewed in organelles that resembled vesiculated mitochondria found in steroid producing cells of the adrenal cortex and testes [11-13].

#### Estrogen effects on mitochondria morphology

The impact of estrogen on mitochondrial morphology has previously been reported in the human breast cancer cell line MCF7. Transmission electron microscopy (TEM) revealed that an 8 day treatment of MCF7 cells with E2 (10 nM) resulted in large, clear mitochondria [14]. These alterations in mitochondria structure were observed as early as 2 days after treatment with a physiologically relevant dose of estrogen. The delamellated cristae of E2 treated mitochondria resemble an early anaerobic state of mitochondria development seen in embryonic rats and primates in which the cell depends on glycolysis [15]. However, it is not known whether the reported estrogen induced morphological changes had an affect on mitochondrial function.

#### Estrogen receptor localization in mitochondria

The function of estrogen at the mitochondria is not clear, however, recent studies have identified ERα and ERβ within the mitochondria implicating its role in the regulation of mitochondrial genome transcription. Subcellular fractionation of rabbit ovarian and uterine tissue revealed isoforms of ERα and ERβ in the mitochondrial enriched fraction as detected by western Blot analysis using ER specific antibodies [16]. More recently, ERβ was shown to be localized in the mitochondria of human lens epithelial cells (HLE-B3), human heart, rat primary neuron and primary cardiomyocyte, and in a murine hippocampal cell line HT-22 [17,18]. Western blot analysis using polyclonal antibodies against human ERα and -β showed both ERs present in purified mitochondria isolated from the human breast cancer cell line MCF7 [19]. Using immunohistochemistry with confocal microscopy and immunogold electron microscopy, ERα and ERβ were identified in the MCF7 mitochondrial matrix. Mitochondrial ERα and ERβ were reported to account for 10% and 18%, respectively, of total cellular ER-α and -β when MCF7 cells were treated with E2. Treatment of MCF7 with E2 (10^-8 ^M and 10^-9 ^M) significantly increased the mitochondrial level of ERα and ERβ by 2.5-fold.

#### Estrogen influence on mitochondrial gene expression

An increasing body of evidence has shown that mitochondrial transcription is enhanced by estrogen treatment. For instance, a 16-fold increase in cytochrome oxidase II (CO II) mRNA is reported in the GH4C1 rat pituitary tumor cell line when treated for 6 days with E2 (0.5 nM) [20]. The mitochondrial gene for subunit III of cytochrome oxidase (CO III) is induced as early as 3 h following a single dose of E2 in the hippocampus of ovariectomized female rats [21]. Other mitochondrial transcripts have also been reported to increase in the human hepatoma cell line, HepG2, and rat hepatocytes when exposed to ethinyl estradiol (EE). A 40 h exposure to an EE concentration ranging from 0.5 to 10 μM resulted in a 2- to 3-fold induction of CO I, CO II, and NADPH dehydrogenase subunit 1 (NADPH-DH1) mRNA [22]. E2 (20 μM), although less potent than EE, showed a similar effect of induction from 1.5- to 1.8-fold in mitochondrial transcripts CO I, CO II, and NADPH-DH1 when treated for 12 h. The E2 catechol metabolite 4-OH-E2 caused a greater response in CO I and CO II transcript levels as compared to E2 after 24 h of treatement with a dose of 10 μM. The mitochondrial gene for ATP synthase subunit 6 (ATPase 6) was also elevated in female rat liver tissue exposed to EE (5 μg/day) for 42 days. An increase in the transcript level of COX7RP (cytochrome c oxidase subunit IV-related protein) was reported after a 6 h E2 (100 nM) treatment in MCF7 cells [23]. It is not known whether the COX7RP transcript is translated to a functional protein in the mitochondria, but the study proposed that COX7RP may represent a regulatory subunit of cytochrome c oxidase that modulates a high state of energy production in estrogen sensitive target tissues. More recently, a 12 h E2 (0.3 μM) treatment of MCF7 cells was demonstrated to enhance the mitochondrial transcript levels of CO I approximately fourfold and CO II~2.5-fold [19].

The mechanism of estrogen-induced mitochondrial gene transcription is not clearly understood. The involvement of estrogen responsive elements (EREs) and/or the ER may be a possible mechanism in the increase of these mitochondrial transcripts. Sequences with partial similarity to the ERE consensus sequence, (AGGTCANNNTGACCT), have been reported in the mouse mitochondrial genome [24]. These partial EREs were detected in genes CO I and CO II which may account for the observed increases in these two transcripts in rat GH4C1 pituitary cells and rat hepatocytes [20,22]. Other genes in which the various EREs were detected include 12S rRNA, 16S rRNA, tRNA-gln, cytochrome oxidase b, unidentified reading frame (URF) 4, URF5, and the D-loop region [24]. In the human mitochondrial genome, we identified partial or ERE 1/2 sites in the D-loop region, CO II, tRNA-met, 12S rRNA, 7S rRNA, URF1, and URF5 (unpublished Felty Q and Roy D). The presence of these partial EREs in the mitochondrial genome may lend support to a novel ER signal transduction pathway. A mechanism of ER translocation into the mitochondria and ER binding to mitochondrial EREs remains unclear. Using electrophoresis mobility shift assay (EMSA) and plasmon resonance analysis, the recombinant human ERα- and ERβ-containing mitochondrial proteins were demonstrated to specifically bind putative EREs in the mtDNA D-Loop, and this ER binding was enhanced by E2 treatment and inhibited by ICI 182780 [19]. Based on this evidence, it is biologically plausible that ER mediates mitochondrial transcription in the same manner as the glucocorticoid receptor (GR) which is translocated into the mitochondria and binds glucocorticoid response elements (GRE) after treatment with glucocorticoid [25,26]. Whether estrogen-induced mitochondrial transcription participates in the development and growth of estrogen dependent breast cancer is not known. Long-term stilbene estrogen (diethylstilbestrol = DES) treatment of Syrian hamsters produced tumors in the kidney with a 5- to 10-fold higher transcript level of CO III than age-matched control kidneys [27].

### Estrogen and the electron transport chain

Besides transcription of mitochondrial genes, estrogen has also been demonstrated to effect mitochondria at the protein level. Estrogen has been demonstrated in several studies to inhibit mitochondrial respiratory complex I, II, III, IV, and mitochondrial ATP synthase (F_0_F_1_-ATPase) [28-31]. Several studies have reported estrogen specific inhibition of mitochondrial respiratory proteins, but it is not clear whether estrogen can modify mitochondrial proteins at the post-translational level. However, there is a report that E2 increased the phosphorylation of a 76 kDa protein in the mitochondrial fraction of the rat corpus luteum [32]. The presence of protein kinases within the mitochondria together with evidence for estrogen-induced phosphorylation of mitochondrial proteins suggest that estrogen may regulate mitochondrial respiratory physiology at the post-translational level [33]. Besides a direct interaction with mitochondrial proteins, estrogen may indirectly effect the electron transport chain (ETC) through an increase of membrane fluidity. Given that thyroid hormone increases mitochondrial membrane fluidity [34] and that physiologic concentrations of estrogen can alter the fluidity of human red blood cell membranes [35], it is likely that a low dose of estrogen may facilitate electron transfer by increasing respiratory protein interactions through more membrane fluidity. Thus, the rate of electron transfer may increase as a consequence of more frequent collisions/interactions between respiratory chain complexes and electron carriers. The three proton pumps of the ETC depend on electron flow to generate the mitochondrial membrane potential (ΔΨ_m_). In human neuroblastoma cells, the following ER ligands: tamoxifen (30.2 μM), clomiphene (10.6 μM), and nafoxidine (2.8 μM); were reported to modulate ΔΨ_m _while E2 did not change ΔΨ_m _at concentrations up to 100 μM [36]. Assuming that estrogen can effect the fluidity of the inner mitochondrial membrane, a rise in ΔΨ_m _could result from an increased rate of electron transfer. The formation of ROS in mitochondria is reported to occur at high ΔΨ_m _[37,38] and therefore suggests that estrogen modulation of ΔΨ_m _may be a possible mechanism for the generation of ROS. Whether physiologic concentrations of estrogen can increase ΔΨ_m _in target tissues is not clear, but these lines of evidence suggest that estrogen may modulate ΔΨ_m _in a dose- and tissue-specific manner. This effect is significant because estrogen-induced mitochondrial reactive oxygen species (ROS) may participate in cell signaling. In the following section, we provide a detailed review of the effects of E2 on mitochondrial respiratory complexes.

#### NADH dehydrogenase (complex I)

The effect of natural estrogens and synthetic estrogens on the mitochondrial ETC has been demonstrated in several studies. Human NADH dehydrogenase (complex I) is the largest respiratory chain complex consisting of 7 mitochondrial genome encoded subunits and more than 41 subunits encoded from the nuclear genome. This integral membrane protein is located within the mitochondrial inner membrane and the matrix. Two electrons enter the ETC from the oxidation of NADH by ubiquinone (CoQ) at complex I which is coupled with proton movement across the inner membrane from the matrix to the intermembrane space. Natural estrogens, 17-α-estradiol, E2, and estrone, at concentrations of approximately 10 μM were demonstrated to inhibit mitochondrial electron transport in homogenates of rat uterus, liver, and skeletal muscle [39]. The synthetic estrogen DES was also reported to inhibit electron transfer from complex I to CoQ at a half-maximal inhibitory concentration range of 0.2–2.6 μM [28]. Although DES at a dose of 20–30 μM could inhibit electron transfer by 90% it was much less effective than rotenone and piericidin A which could inhibit electron tranfer by 90–95% at a dose of 30–50 nM due to a tighter binding affinity to complex I. The researchers postulated that at relatively low doses, DES reversibly inhibits electron transfer at complex I. Additionally, DES displayed specific binding to a site in which rotenone and piercidin A bind to complex I. Since photoreactive analogues of rotenone have been reported to label complex I [40], these results implicate estrogen specific binding to respiratory complex I. Another class of compounds, phytoestrogens, which are found in our diet can also inhibit the activity of complex I. Phytoestrogens, genistein (found in soy beans) and resveratrol (found in red wine), can inhibit the activity of complex I, but are considered less active than rotenoid compounds [41].

There are very few reports that have investigated co-treatment of estrogen with mitochondrial inhibitors of oxidative phosphorylation (OXPHOS). Complex I inhibitors rotenone, piericidin A, and amytal have been used in co-treatment with DES and/or estrogens to elucidate the site of estrogen action on electron transfer [28,39]. In MCF7 cells, it was demonstrated that co-treatment with rotenone (10 nM) and E2 (10 nM) strongly inhibited ornithine decarboxylase activity by 86% [42]. More recently, a study reported that treatment with 10 μM of 2-methoxyestradiol (2-Me) induced apoptosis in Ewing sarcoma cells through hydrogen peroxide (H_2_O_2_) production [43]. Since a 2 h prior treatment of rotenone (6 μM) inhibited 2-Me induced apoptosis and H_2_O_2 _production, it was suggested that the H_2_O_2 _source was the mitochondria. Although the biological significance of estrogen interaction with complex I is unknown, evidence of estrogen inhibition of electron transfer support a novel role of estrogen in the formation of mitochondrial ROS.

#### Succinate dehydrogenase (complex II)

Human succinate dehydrogenase (complex II) is a membrane bound protein located on the matrix side of the inner mitochondrial membrane. Complex II consists of 4 subunits all encoded from the nuclear genome. Two electrons enter the mitochondrial ETC from the oxidation of FADH_2 _by CoQ at complex II. Although previous studies have reported complex I and cytochrome bc_1 _reductase (complex III) as the major sites of ROS generation, ubiquinone radicals have been reported to contribute to basal levels of ROS at complex II [44]. Physiologically relevant ROS generation supported by the complex II substrate succinate occurs at complex I through reversed electron transfer [45]. Research studies of the protective effect of estrogen on the brain use the complex II inhibitor, 3-nitroproprionic acid (3-NPA) to model the condition of ischemia. Although the mechanism of estrogen neuroprotection is not clearly understood, estrogen has been proposed to modulate cerebral energy/glucose metabolism. Both 17-β-E2 and 17-α-E2 have been demonstrated in vivo to reduce ischemic brain damage induced by middle cerebral artery occlusion in ovariectomized rats [46,47]. To model an in vitro state of interrupted energy metabolism as seen in cerebral ischemia and chronic neurodegenerative disease, the human neuroblastoma cell line SK-N-SH was treated with 3-NPA (10 mM) [48]. This study demonstrated that pretreatment of SK-N-SH with E2 (2 μM) restored ATP levels to 80% at 12 h as compared to the control cells treated with 3-NPA alone. Whether mitochondrial function can be preserved with a physiological concentration of estrogen is not clear as this study used a high dose (2 μM) which can be cytotoxic in certain tissues. The maintenance of ΔΨ_m _and ATP levels by estrogen when faced with 3-NPA toxicity was proposed to be due to the antioxidant effect and/or ATP increasing effects. At the 2 μM E2 dose an antioxidant effect is likely because estrogen is reported to be an effective neuroprotective antioxidant in the micromolar dose range [49]. However, another possibility may be due to an estrogen-induced increase in complex II activity which would overcome inhibition by 3-NPA as succinate dehydrogenase activity has been reported to increase in the brain of E2 treated rats [29]. Estrogen has been shown to cause multiple effects at the level of complex II that include the inhibition of electron transfer, maintenance of ΔΨ_m _and ATP levels, and the enhancement of succinate dehydrogenase activity.

#### Cytochrome bc_1 _reductase (complex III)

The human cytochrome bc_1 _reductase (complex III) is an integral membrane protein located in the inner mitochondrial membrane consisting of 10 subunits encoded from the nuclear genome and 1 subunit encoded from the mitochondria. Complex III transfers electrons from ubiquinol (CoQH_2_) to cytochrome c which is coupled to proton movement across the inner membrane. The synthetic stilbene estrogen, DES (10 μM–50 μM), is reported to inhibit complex III at the site of electron flow from ubiquinone to cytochrome c_1 _[50]. The ER ligand tamoxifen is also reported to inhibit electron transfer at the site of complex III in isolated rat liver mitochondria [30]. The phytoestrogen resveratrol has been reported to bind both ER α/β, increase expression of estrogen responsive genes, and stimulate cell proliferation of MCF7 and T47D breast cancer cells [51,52]. Interestingly, resveratrol has been shown to inhibit complex III activity (20%) by competition with ubiquinol (CoQH_2_) and preserve mitochondrial function by its action on complex III in isolated rat brain mitochondria [53,54]. Besides the inhibition of electron transfer at complex III, the phytoestrogen genistein (50 μM) induced mitochondrial permeability transition (MPT) in isolated rat liver mitochondria. Since estrogen can inhibit electron transfer at complex III, the potential of estrogen to modulate the formation of mitochondrial ROS exists at multiple respiratory complexes.

#### Cytochrome c oxidase (complex IV)

In humans, the mitochondrial cytochrome c oxidase (Complex IV) is located in the inner membrane and catalyzes the transfer of electrons to oxygen with water being the final product of the reduction reaction coupled with proton movement across the inner membrane. The mitochondria genome encodes 3 subunits of complex IV while the other 10 subunits are encoded from the nuclear genome. The effect of estrogen and ER ligands on complex IV has been reported to increase and decrease enzymatic activity. During the oestrus cycle in rats, E2 decreased complex IV activity in brown adipose tissue, which suggests a role for E2 in the modulation of oxidative capacity [55]. The ER ligand tamoxifen was reported to restore complex IV activity to normal levels in disrupted rat liver mitochondria [56]. In rat liver mitochondria, tamoxifen was demonstrated to have a biphasic effect on complex IV in which a low concentration of 10–15 μM increased enzyme activity while a higher concentration of 50 μM inhibited activity by 50% [30]. Although the biological effect of estrogen actions on complex IV remains to be elucidated, it has been suggested that estrogen modulation of complex IV activity may increase energy production in estrogen sensitive tissues [23]. Furthermore, it has been proposed that ΔΨ_m _and ROS formation may be controlled by a hormone mediated reversible phosphorylation of complex IV [57]. Thus, the possibility for estrogen control of ROS formation by modulating complex IV activity may provide a mechanism of estrogen-induced redox signaling by the mitochondria.

#### Mitochondrial ATP synthase (F_0_F_1_-ATPase/Complex V)

Mitochondrial ATP synthase (F_0_F_1_-ATPase/Complex V) is composed of two distinct parts: 1) the F_1_-ATPase portion which protrudes into the matrix and synthesizes ATP when protons pass through it down their electrochemical gradient. 2) the F_0_-ATPase which forms a transmembrane proton channel through the inner membrane. F_0_F_1_-ATPase is encoded by 2 mitochondrial genome encoded subunits and 14 nuclear encoded subunits. F_0_F_1_-ATPase like respiratory chain complexes I-IV represents another enzyme in the mitochondrial inner membrane that is sensitive to estrogen. Inhibition of rat liver F_0_F_1_-ATPase by DES (10 μM) has been demonstrated and the F_0 _portion was reported to contain a distinct binding site for DES [58]. This specific binding site for the F_0 _portion has made DES a unique probe for the rapid isolation of functional F_0 _from rat liver mitochondria [59]. Using E2-BSA conjugates, a 23 kDa estrogen binding protein was identified in rat brain mitochondria [60]. The 23 kDa protein was identified as the oligomycin-sensitivity conferring protein (OSCP) which forms the stalk region between F_0 _and F_1 _subunits of F_0_F_1_-ATPase in mitochondria. The OSCP was proposed to be the specific site on F_0_F_1_-ATPase where estrogen modulates ATPase activity. Differential effects of estrogen on F_0_F_1_-ATPase activity in isolated rat heart, liver, and brain mitochondria were observed when treated with E2, DES, and resveratrol [31]. E2 (13 nM) stimulated F_0_F_1_-ATPase activity in the heart by 10%, but not in the liver and brain. The phytoestrogen resveratrol (13–15 μM) inhibited F_0_F_1_-ATPase activity in the heart and liver while lower doses (133 pM–1.3 nM) stimulated F_0_F_1_-ATPase activity in the liver by 10%. Both phytoestrogens resveratrol (19 μM) and genistein (55 μM) can inhibit F_0_F_1_-ATPase activity in rat brain mitochondria [61]. In rat heart, liver, and brain the mitochondrial F_0_F_1_-ATPase activity was inhibited by DES (6.7 μM) 61%–67% [31]. In rat brain mitochondria, 17α-estradiol and E2 partly inhibited F_0_F_1_-ATPase activity at low concentrations of 15 nM and 3.4 nM, respectively, while 17α-E2 preserved mitochondrial function altered by the stress of anoxia-reoxygenation [62]. Although these studies of estrogen effects on F_0_F_1_-ATPase have been shown in isolated mitochondrial preparations, F_0_F_1_-ATPase inhibition by E2 was demonstrated to occur with intact human osteolclastic FLG 29.1 cells [63]. These studies of F_0_F_1_-ATPase activity demonstrate that estrogens and estrogen-like compounds possess cell-type and dose-specific effects on mitochondrial function.

### Modulation of mitochondrial ROS by estrogens

Within the cell, mitochondria are considered to be a major source of ROS, which include superoxide anion (O_2_^•-^), H_2_O_2_, and the hydroxyl free radical (^•^OH) [64-66]. Since mitochondria consume 85% of the oxygen used by the cell, the mitochondrial ETC generates a substantial amount of intracellular ROS [67]. As electrons pass through the mitochondrial ETC, some electrons leak out to molecular oxygen (O_2_) to form O_2_^•- ^which is dismutated by manganese superoxide dismutase (MnSOD) to form H_2_O_2 _[64,68]. During mitochondrial respiration, 2% of the electron flow is reported to result in the formation of H_2_O_2 _[66]. However, lower values of free radical leak were reported in the range of 0.4%–0.8% for heart mitochondria respiring on physiological concentrations of succinate (<0.5 mM) [37]. In support of these findings, intact rat skeletal muscle, heart, and liver mitochondria were reported not to produce measurable amounts of ROS when respiring on complex I and complex II substrates [69]. In addition, this study reported a lower estimate of electron flow (0.15%) that contributed to H_2_O_2 _production under resting conditions. These results suggest that mitochondria produce low levels of ROS that can be effectively scavenged by the cell's antioxidant defenses at resting conditions. It is this point, the low basal level of ROS produced by the mitochondria at rest, which makes mitochondrial ROS ideal signaling molecules since its contribution to the intracellular level of ROS is not at so high a level to induce oxidative stress; instead, a low oxidant level provides a physiologically safe window for redox signaling which allows the cell to regulate mild to moderate oxidative changes and critically respond to them by activating cellular processes such as proliferation and differentiation rather than triggering cell death.

#### Characteristics of mitochondrial ROS

Mitochondria are a predominant source of ROS in most cell types with unique characteristics that may allow it to participate in growth signal transduction. First, mitochondria are unique because they are a regulatable source of ROS in response to external stimuli. For example, cortical neurons exposed to N-methyl-D-aspartate (NMDA) were reported to couple a rise in intracellular calcium with mitochondrial O_2_^•- ^production [70]. Tumor necrosis factor alpha (TNF-α) is another example of stimulated mitochondrial generation of O_2_^•- ^in L929 cells and this ROS generation is coupled to the cytokine by the TNF-α receptor [71,72]. Few other examples exist of mitochondria producing ROS in response to external stimuli, but more recently integrins (cell surface receptors that interact with the extracellular matrix) were reported to modulate mitochondrial ROS production for signal transduction [73]. Although signal pathways involved in triggering mitochondrial ROS remain largely unknown, it has been proposed that mitochondria participate in integrin signaling in a nonapoptotic manner, which leads to gene expression and cell differentiation.

Mitochondrial ROS can enter the cytosol as either H_2_O_2 _or O_2_^•- ^where it can participate in redox signaling. Within the mitochondria, MnSOD can dismutate O_2_^•- ^to H_2_O_2 _which is a highly diffusible signaling molecule that can exit the mitochondria. In addition to H_2_O_2_, O_2_^•- ^was demonstrated to be released by mitochondria to the cytosol via the voltage-dependent anion channels (VDACs) [74]. In regard to turning the mitochondria ROS signal off, cellular antioxidant defenses such as SOD, catalase, and glutathione peroxidase easily degrade ROS, which terminates the signal. Therefore, mitochondrial ROS fulfill the prerequisites of a 2^nd ^messenger since they are short-lived (rapidly generated and degraded), produced in response to a stimulus, highly diffusible, and ubiquitously present in most cell types.

Mitochondria are highly dynamic structures capable of changing their shape (by elongation, branching, swelling) and their location inside a living cell [75]. It is becoming clear that the morphological, functional, and genetic differences (heteroplasmy) that exist within the mitochondria population may reflect a division of labor within the cell. Mitochondria have been reported to be morphologically heterogeneous and unconnected within individual cells [76]. Pancreatic acinar cells were reported to contain distinct groups of mitochondria classified by their cellular location that included perinuclear, subplasmalemmal, and perigranular mitochondria [77]. In light of this finding, the highly diffusible H_2_O_2 _generated by mitochondria may become a specific signaling molecule as a function of location. For example, perinuclear mitochondria may generate H_2_O_2 _that only transduces signals to the nucleus or the subplasamlemmal mitochondria may only activate signal cascades of plasma membrane origin. Additionally, perinuclear, subplasmalemmal, and perigranular mitochondria were independently activated by intracellular calcium signals in their immediate environment, which supports distinct calcium functions for each type of mitochondria.

It is significant that mitochondria can create subcompartments or 'microzones' within the cytoplasm because signal transduction depends on the close proximity of substrates and effector molecules to be an efficient process. In addition, given the presence of other endogenous ROS sources besides the mitochondria such as NADPH oxidase, peroxisomes, cytochrome p450, xanthine oxidase, cyclooxygenase, lipooxygenase, and γ-glutamyl transpeptidase [78]; and because ROS is involved in a variety of signal cascades, understanding how mitochondrial ROS is activated at the right place and at the right time is vital in understanding the organelle's role in signal transduction. Compartmentalization has already been reported to play a key role in redox signaling and we consider this attribute when describing the mitochondria as a signal transducer [79]. In adult cells, mitochondrial clustering functions to create steep gradients of low molecular weight species such as O_2_, ATP, and pH resulting in specialized microzones that may facilitate signal specificity [80]. In the cytosol, the volume occupied by mitochondria in cells is highly variable and ranges from 15% to 50%. Based on volume, mitochondria compose a significant compartment within the cytosol that harbors signaling molecules. H_2_O_2 _produced within the mitochondria is highly diffusible in contrast to O_2_^•-^, which cannot diffuse through membranes making it easily compartmentalized. Thus, mitochondrial generated O_2_^•- ^may be kept separated from the cytosol until an appropriate stimulus releases it through VDACs. Another route for O_2_^•- ^release may be through the mitochondrial permeability transition pore (MPTP) as low molecular weight compounds up to molecular weight 1500, can be exchanged between the mitochondrial matrix and the cytosol via this pore [81]. Since the MPTP is reported to reversibly open/close naturally in intact cells without resulting in apoptosis, mitochondrial signaling molecules could be exchanged with the cytosol by the transient 'flickering' (open/closing) of the MPTP in response to certain stimuli [82].

In addition to location and compartmentalization, protein scaffolds mediate the selective activation of the mitogen activated protein kinase (MAPK) signaling pathway which raises the question of whether mitochondria may also act as a protein scaffold for signaling complexes [83]. A-kinase anchoring protein (AKAP) is reported to tether protein kinase A (PKA) to the mouse mitochondrial outer membrane [84,85]. What makes AKAPs unique is their ability to simultaneously bind multiple signaling enzymes such as other kinases and phosphatases [86]. This multivalent scaffold has been described as a 'transduceosome' capable of integrating signals from multiple pathways [87]. Whether these multivalent scaffolds exist on mitochondria is not clear at this time, but these signaling complexes could be a mediator of signals between the mitochondria and the nucleus during cell division. For example, AKAP84/121 has been demonstrated to concentrate on mitochondria in interphase and on mitotic spindles during metaphase transition alluding to its role in the cell cycle [88]. In addition to AKAP, a mitochondrial signaling complex has been reported to activate MAPKs. The PKCε can form signaling complexes with ERKs, JNKs, and p38 MAPKs in the murine heart [89]. Activated PKCε was shown to increase phosphorylation of mitochondrial ERK and p38 MAPKs. Whether the anchoring of PKC to mitochondria depends on AKAP is not known at this time.

#### Mechanisms of estrogen-induced mitochondrial ROS

Studies of the mitochondrial ETC have reported only two ROS forming sites, the FMN group of complex I and the ubiquinone site in complex III [45,90]. The topology of ROS production, on which side of the mitochondrial inner membrane O_2_^•- ^is produced, was reported to occur on the cytosolic side by complex III and on the martix side of the inner membrane by complex I [69]. Estrogen is known to act as either an antioxidant or pro-oxidant depending on the concentration [91]. Whether physiological concentrations of estrogen can stimulate mitochondrial ROS at complex I and complex III is not clear since most studies have been performed with cytotoxic doses. In the following section, we provide evidence for potential mechanisms of estrogen induced mitochondrial ROS.

Electrons feed into the mitochondrial ETC at complex I and complex II. At complex I and complex II, ubiquinone or co-enzyme Q_10 _(CoQ) oxidizes NADH and FADH_2_, respectively. CoQ functions as a mobile electron carrier within the mitochondrial inner membrane and transfers 2 electrons from both NADH and FADH_2 _to complex III [92]. CoQ is known to offer protection from heart disease by increased ATP production and antioxidant actions [93]. It is a highly lipophillic compound due to its structure which includes an isoprenoid tail of usually 10 isoprene units in length, hence the designation Q_10_. Other than its role as an electron carrier and antioxidant, CoQ is also reported to act as a pro-oxidant. Although the pro-oxidative action of CoQ within the mitochondria is a matter of debate, O_2_^•- ^formation occurs from a single electron transfer from ubisemiquinone to molecular oxygen. Exogenously added CoQ has been demonstrated to enhance O_2_^•- ^generation in isolated respiratory complex I and III [94]. Further evidence in support of CoQ redox-cycling come from a study that demonstrated H_2_O_2 _derived from decomposing O_2_^•- ^was inhibited after the removal of CoQ [90]. Upon the re-addition of CoQ, H_2_O_2 _was detected again which indirectly demonstrated that the O_2_^•- ^may originate from CoQ. Although some reports make a case for O_2_^•- ^formation by CoQ redox cycling in mitochondria, arguments against its role as a source of O_2_^•- ^come from a study which demonstrated that O_2_^•- ^formation did not occur in a water-free nonpolar reaction system that mimics the lipophilic nature of the inner mitochondrial membrane [95]. However, pretreatment of the membrane with toluene which increased its permeability to protons provided conditions in favor of O_2_^•- ^formation by CoQ. Thus, it was proposed that under certain pathological conditions in which the inner mitochondrial membrane is protonated, CoQ becomes a significant source of O_2_^•- ^[96]. In line with this result, a study demonstrated that CoQ (100 μM) enhanced the release of H_2_O_2 _from mitochondria in the presence of antimycin A (2 μM) and to a lesser extent with Ca^2+ ^(10 μM) [97]. The antimycin A and Ca^2+ ^pretreatment was thought to induce a leaky inner mitochondrial membrane thereby allowing protons to interact with CoQ and enhance ROS production by redox cycling.

Interestingly, CoQ shares similar characteristics to catechol metabolites of estrogen. The catechol metabolites 2- and 4-OH-E2 contain hydroxyl groups that can be oxidized to semiquinones, which in the presence of molecular oxygen can be further oxidized to quinones with the formation of O_2_^•- ^[98]. Since CoQ undergoes reduction/oxidation (redox) reactions which result in the radical semiquinone intermediate (semiubiquinone) and quinone, it is biologically possible for catechol estrogens to participate in shuttling electrons and to act as a pro-oxidant like CoQ. Given that MCF7 cells treated with the o-quinone form of estrogen and NADPH produce significant amounts of H_2_O_2 _in the mitochondrial subfraction [99]; and that mitochondrial enzymes catalyze redox reactons of stilbene estrogen in the mitochondria [100], the capacity of catechol estrogens to redox cycle within mitochondria suggests that these metabolites could facilitate mitochondrial ROS formation. Assuming that estrogen is a weak electron carrier compared to CoQ, it may have a tendency to leak electrons to molecular oxygen instead of transferring its electrons to complex III. The phytoestrogen and dietary flavinoid quercetin is reported to act as a pro-oxidant. Foods of plant origin contain flavinoids known to act as antioxidants or pro-oxidants depending on the concentration and metal chelates which catalyze the oxidative process [101]. Estrogen is similar to some flavinoids with respect to inhibition of the mitochondrial respiratory chain at complex I and complex II [102,103]. Based on these reports, the formation of O_2_^•- ^by estrogen at the level of electron transport could be one mechanism of increased mitochondrial ROS.

Mitochondrial Ca^2+^, [Ca^2+^]_m_, accumulation is reported to promote the generation of ROS [104]. For example, an increase in [Ca^2+^]_m _was reported to stimulate H_2_O_2 _production by rat brain mitochondria in the presence of rotenone [105]. Using confocal microscopy, we have shown a time-dependent increase in [Ca^2+^]_m _with E2 (100 pg/ml) treatment of MCF7 cells [106]. Another study reported an increase in [Ca^2+^]_m _with E2 (10 ng/ml) treatment of hippocampal neurons [107]. The mechanism for these increases in [Ca^2+^]_m _is not clear, however, the inhibition of Na-dependent Ca^2+ ^efflux from mitochondria was reported to increase calcium retention in E2 (1 nM) treated synaptosomal mitochondria [108]. There is some evidence which suggests allosteric inhibition of a respiratory complex may be a mechanism for hormone induced ROS formation. Allosteric inhibition of the respiratory complex IV (COIV) or cytochrome c oxidase is reported to occur by cAMP-dependent phosphorylation; and this inhibition is turned-off by Ca^2+^-activated dephosphorylation [109]. A study proposed that a hormone stimulated increase of cellular Ca^2+ ^may activate a mitochondrial protein phosphatase which dephosphorylates cytochrome c oxidase. In turn, cytochrome c oxidase is activated which results in a rise of ΔΨ_m _and ROS [57]. Interestingly, the ER ligand tamoxifen (15 μM) showed a slight stimulatory effect on cytochrome c oxidase [30]. Since estrogen is capable of increasing [Ca^2+^]_m_, it is possible for estrogen to signal the formation of mitochondrial ROS through a similar mechanism.

The inhibition of respiratory complex I is known to favor ROS generation. Rat brain mitochondria that respired on complex I substrates produced a substantial level of ROS when inhibited with rotenone concentrations as low as 20 nM [110]. Since estrogen is known to inhibit respiratory complex I, we speculate that complex I interactions with the hormone could favor ROS production in a manner similar to rotenone. The phytoestrogen genistein is another flavinoid besides quercetin that acts like a pro-oxidant at the level of mitochondria. Genistein (50 μM) treatment of rat liver mitochondria was shown to increase ROS formation through interaction with respiratory complex III which resulted in the opening of the membrane transition pore [111]. Besides hormone interactions with respiratory enzymes, post-translational modifications such as phosphorylation-/-dephosphorylation that affect the activity of mitochondrial proteins should also be considered in ROS generation. The cAMP-dependent protein kinase is reported to phosphorylate 6-, 18-, 29-, 42-kDa mitochondrial proteins in bovine heart and phosphorylate the human 18-kDa subunit which promotes the activity of complex I [112-115]. Since estrogen is reported to stimulate cAMP-dependent protein kinase activity in hippocampal neurons, it raises the possibility for estrogen to induce cAMP accumulation in mitochondria [116,117]. If estrogen increased cAMP levels within mitochondria, then cAMP-dependent phosphorylation of mitochondrial respiratory complexes may modulate ΔΨ_m _and/or [Ca^2+^]_m _in favor of ROS generation.

Several isoforms of the enzyme nitric oxide synthase (NOS) are reported to exist which include inducible-(i), endothelial-(e), neuronal-(n), and mitochondrial-(mt) NOS. Estrogen has been reported to induce various isoforms of NOS. The activity of eNOS is modulated by estrogen in human aortic endothelial cells, uterine artery, heart, and skeletal muscle [118,119]. Estrogen has also been shown to stimulate protein expression of nNOS in human neutrophils and the transcription of iNOS in rat macrophages [120,121]. These effects are not limited to E2 because the estrogen-like chemical bisphenol A and the phytoestrogen resveratrol are also reported to stimulate NO synthesis [122,123]. These lines of evidence demonstrate a significant role of estrogen compounds in the modulation of NOS and NO. In regards to redox signaling, a NO-dependent inhibition of cytochrome c oxidase has been proposed to generate O_2_^•- ^which is dismutated into the membrane permeable second messenger H_2_O_2 _[124]. Since estrogen is capable of inducing NOS activity and expression, we postulate that an estrogen induced rise in NO could participate in a similar manner whereby generating mitochondrial H_2_O_2_. Interestingly, NO induced mitochondrial biogenesis has been demonstrated in several cell lines, which include brown adipocytes, 3T3-L1, U937, and HeLa cells [125]. We have shown that estrogen can influence mitochondrial biogenesis (data unpublished) and postulate that estrogen-induced NO could be one possible mechanism. More specifically, since mtNOS activity is dependent on Ca^2+^, we propose that an estrogen-induced rise in [Ca^2+^]_m _could stimulate mtNOS activity ultimately leading to the generation of ROS via NO-dependent inhibition of cytochrome c oxidase [126].

### Estrogen-induced growth of cells in relation to mitochondria

The rapid stimulation of intracellular ROS by platelet-derived growth factor (PDGF), epidermal growth factor (EGF), and nerve growth factor (NGF) suggests that this underlying mechanism of cell growth may be shared with other growth factors including estrogen [127]. Exogenous addition of low concentrations of H_2_O_2 _and/or O_2_^•- ^has been demonstrated to stimulate cell growth in a variety of cell types including muscle cells, fibroblasts, amnion cells, prostate cancer cells, and aortic endothelial cells [78]. The molecular signaling mechanism that initiates ROS production by mitochondria is not clear, however, other cell processes besides apoptosis may be coupled to this signaling event. Tumor necrosis factor alpha (TNF-α) induces gene expression via mitochondrial respiratory chain dependent activation of NF-κB, AP-1, JNK, and MAPKK [73]. The proliferative response of endothelial cells to hypoxia was demonstrated to be initiated upstream by mitochondrial ROS which activated the MEK/ERK pathway [128]. Although other endogenous ROS sources besides mitochondria such as NAD(P)H oxidase exist, mitochondria will be the focus of this paper for the following reasons: (*i*) mitochondria are the principal source of intracellular ROS in epithelial cells. (*ii*) the growth of adenocarcinomas occur in tissue of epithelial cell origin.

A characteristic of rapidly dividing cancer cells is their capacity to produce significant amounts of intracellular ROS, which has been implicated in the promotion of accelerated cell cycle activity in neoplastic cells. Mitochondria have long been suspected to play a role in the development and progression of cancers. The ROS molecules H_2_O_2 _and NO have been demonstrated to stimulate mitochondrial biogenesis, a process that depends on the flow of molecules into and out of the organelle [125,129]. Since mitochondrial proteins are encoded in two separate genomes (mitochondria and nuclear genome), biogenesis is a coordinated effort in which mitochondria transmit signals to the nucleus and vice versa. The question of how mitochondria transmit these signals in the process of cell proliferation has risen from reports of its involvement in cell growth. Cerebral granular cells isolated from newborn rats with high mtNOS activity were reported to exhibit maximal proliferation rates which depended on NO and H_2_O_2 _levels. In addition, MnSOD displayed an increased pattern of activity similar to mtNOS [130]. NO has been proposed to inhibit cytochrome c oxidase in favor of O_2_^•- ^production and therefore MnSOD may dismutate O_2_^•- ^generated by NO-dependent inhibition into the signaling molecule H_2_O_2_. Ethinyl estradiol, E2, and estrogen catechol metabolites at a dose of 0.25 to 5 μM are reported to increase mitochondrial O_2_^•- ^in cultured rat hepatocytes and HepG2 cells [131]. Although the biological significance of the estrogen-induced mitochondrial O_2_^•- ^is not known at this time, ROS has been demonstrated to modulate ER protein expression in various cell lines. Treatment of human breast cancer cells MCF7 and T-47D with H_2_O_2 _(2.5 μM) increased the protein level of ERβ [132]. In addition, PMA (100 ng/ml) treatment increased the expression of ERβ in the macrophage cell line J774A.1.

Evidence for the involvement of redox signaling with estrogen-induced cell proliferation has been demonstrated in several studies. Liposomes containing SOD or catalase inhibited *in vitro *estrogen-induced proliferation of Syrian hamster renal proximal tubular cells [133]. The cytokines IL-1β and TNF-α are known to cause the release of O_2_^•- ^from human fibroblast cells. Co-treatment with an inhibitor of IL-1β and TNF-α synthesis, pentoxifylline, inhibited stilbene estrogen-induced increase in myeloperoxidase activities, 8-hydroxydeoxyguanosine (8-OHdG) formation, mutations in the testicular genome, and prevented estrogen-induced testicular preneoplastic lesions [3]. Recently, we have shown that estrogen-induced stimulation of macrophage cells and MCF7 cells in part occurs through ROS [134,135]. We have also observed inhibition of estrogen-induced MCF7 cell growth by ROS scavengers such as N-acetylcysteine, ebselen, and catalase (unpublished Singh M, Felty Q and Roy D). ROS can modulate effector molecules such as PKC, p53, extracellular regulated kinase (ERK), nuclear factor-κB (NF-κB), and the c-fos/c-jun heterodimer (AP-1); and these effector molecules are known to participate in growth signal transduction [136]. Therefore, estrogen-induced production of mitochondrial ROS may activate cell growth in estrogen-sensitive tissues.

Oxidative stress has been shown to affect mitochondrial proteins of chronically estrogenized Syrian hamster kidney. A decrease in thiol/sulfhydryl groups was reported in the mitochondrial fraction at a preneoplastic stage of carcinogenesis [137]. Estrogen-induced oxidative stress may be responsible for these post-translational modifications in mitochondrial proteins. This finding is significant in the context of cell signaling because redox reactions involving cysteine thiol groups transduce signals by breaking or forming protein dithiol/disulfide bridges [138]. Since estrogen can induce mitochondrial ROS, we infer that the oxidation of thiols in response to estrogen converts the oxidative stress to a change in protein function involved in cell growth. Oxidative stress modifies mitochondrial matrix protein thiols [139]. Similarly, thiols on protein subunits 51-kDa and 75-kDa of NADH dehydrogenase (complex I) have been reported to form mixed disulfides with glutathione (glutathionylation) in response to mitochondrial oxidative stress. This post-translational modification was reversible and correlated with an increase in mitochondrial O_2_^•- ^production [140]. Evidence in support of a ROS signal transduction pathway originating from complex I comes from a study which reported that the mitochondrial complex I inhibitor, rotenone, blocked ROS mediated signaling. Interestingly, this study demonstrated that a co-treatment of rotenone (10 nM) and E2 (10 nM) inhibited ornithine decarboxylase activity by 86% in MCF7 cells [42]. Since ornithine decarboxylase activity is a marker for cell growth, it appears that a signal transduction pathway for estrogen-induced cell growth may originate from the mitochondria assuming that rotenone inhibition is specific to complex I. The antitumor arotinoid, mofarotene (Ro 40-8757), has been demonstrated to down-regulate mitochondrial encoded NADH dehydrogenase subunit 1 (MtND1) expression in breast cancer cell lines MDA-MB-231, ZR-75-I, and MCF7 [141]. Since MtND1 has been reported to form part of the rotenone-binding site in complex I [40], the absence of MtND1 may remove an important site of estrogen action in mofarotene treated cells and may account for the anti-proliferative effects of this compound. Whether these protein interactions and/or modifications can occur as a result of estrogen exposure remains to be investigated. From these investigations, we infer that estrogen mediated cell growth via mitochondrial generated ROS signaling molecules may exist and merits future exploration to address this novel pathway.

The mitochondrial thioredoxin system has been demonstrated to play a role in cell cycle progression. In general, the two antioxidant oxidoreductase enzymes thioredoxin (Trx) and thioredoxin reductase (TrxR) that compose the system modulate signal transduction properties of ROS by the reduction of intracellular disulfides. Trx acts as a protein disulfide reductant for ribonucleotide reductase and several transcription factors including p53, NF-κB, and AP-1 [142]. Once oxidized the active disulfide site is reduced by TrxR re-generating the reductant form of Trx. Enzyme isoforms Trx-2 and TrxR2 are reported to exist in the mitochondria. A biological role for TrxR2 in cell growth was demonstrated in HeLa cells using a dominant negative form of TrxR2 (TrxR2DN) [143]. An increase of G_1 _to S phase transition, cell growth, and transcription of cell cycle genes was induced by TrxR2N expression. TrxR2DN expression was suggested to increase intracellular H_2_O_2_, which in turn signaled cell proliferation. Although it is not clear whether estrogen can modulate the H_2_O_2 _levels of mitochondria, estrogen (10 nM–100 nM) treatment of primary human endometrial stromal cells *in vitro *show an increase in Trx protein and mRNA which implicate Trx involvement in cell growth and differentiation of estrogen responsive tissue [144]. Alterations in cellular redox status by increased expression of TrxR2 have been suggested to play a role in the growth of hepatocellular carcinomas [145]. Whether estrogen can signal cell growth through Trx2 and/or TrxR2 is not known, but these findings suggest that estrogen may modulate signal transduction of mitochondrial derived ROS via the thioredoxin system.

### Transduction of estrogn-induced mitochondrial signals to nucleus

Mitochondrial ROS fulfill the characteristics of a 2^nd ^messenger since they are short-lived (rapidly generated and degraded), produced in response to a stimulus, highly diffusible (H_2_O_2_), and ubiquitously present in most cell types. It is not known whether mitochondrial ROS like H_2_O_2 _are involved in signaling pathways that control estrogen-induced cell proliferation. In this section, we provide evidence in support of redox signaling pathways of mitochondrial origin, which may be involved in cell cycle progression of estrogen-dependent cells.

#### Redox sensor proteins and transcription

**Figure 1:**
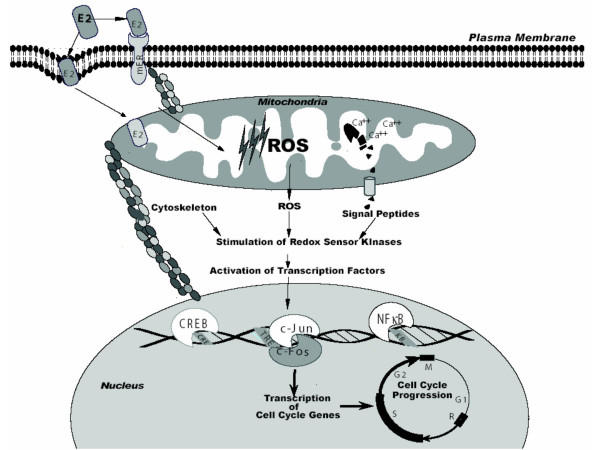
Hypothetical scheme outlining three E2-induced signaling pathways from mitochondria. (*i*) E2 binding to a plasma membrane receptor and/or mitochondrial respiratory complexes generates ROS which leads to kinase activation. (*ii*) E2-induced rise in mitochondrial calcium leads to the activation of calcium-dependent proteases which process signal peptides, in turn responsible for kinase activation. (*iii*) E2-induced cytoskeleton modifications by mitochondria leads to kinase activation. Increased kinase activity results in the activation of transcription factors responsible for cell cycle progression.

Protein kinases are known to participate in phosphorylation signal cascades, however, zinc finger domains contained in some proteins may allow them to participate in redox signaling networks. Zinc finger structures within a protein consist of at least two zinc-coordinated thiolates. Upon oxidation zinc is released from the protein, which converts cysteine thiol groups to disulfide. A conformational change in the protein may result in either its activation or inhibition. There are several protein kinases such as a-raf and PKC that contain zinc finger domains. In addition, zinc finger domains are also found in hormone receptors such as the GR and ER [146]. Both PKC and c-raf have been demonstrated to be redox activated at the zinc finger domain [147,148]. For instance, ROS can trigger the release of zinc ions from PKC which results in its activation. Another protein kinase, c-raf, known to participate in the MAPK signal cascade was also demonstrated to be redox activated at the zinc finger domain. The mitochondrial localization of protein kinases src, Akt, a-raf, and PKC is evidence that this subcellular compartment harbors oxidant sensitive proteins that may facilitate cross-communication between redox and phosphorylation networks [33,89]. Although the role of the protein kinase a-raf in the mitochondria is not clear, a-raf mRNA is highly expressed in normal murine tissues such as the epididymis, ovary, kidney, and urinary bladder [149]. In Hela cells, epidermal growth factor rapidly (2 min.) and transiently activated a-raf, which in turn phosphorylated the MAP kinase activator MEK1 [150]. Therefore, mitochondrial ROS may activate MAPK signaling via a-raf. It is interesting to note that E2 can stimulate the phosphorylation of a-raf and cell cycle progression in MCF7 cells [151]. Whether the estrogen induced phosphorylation of a-raf depends on ROS is not known. Mitochondrial PKCδ and PKCε could also activate the raf/MEK/ERK pathway or directly activate MAPKs, respectively [152,153]. Rapid effects of estrogen have been demonstrated to mediate the DNA binding activity and phosphorylation of transcription factors. E2 treatment of rat adipocytes doubled AP-1 DNA binding and phosphorylated CREB protein within 15 min [154]. The redox sensitive protein Akt is known to phosphorylate an upstream kinase, IKKα, which stimulates the degradation of Iκ-B [155]. Estrogen-induced mitochondrial ROS may stimulate Akt leading to the degradation of Iκ-B and activation of the transcription factor NF-κB. Whether estrogen treatment can activate Akt via mitochondrial derived ROS is not clear, however, phosphorylation and translocation of Akt to the mitochondria was demonstrated when cells are treated with estrogen [156]. Given that E2 can stimulate mitochondrial ROS generation; ER, src, a-raf, Akt, and PKC are targets of oxidative stimuli localized at the mitochondria; and the transcription factors AP-1, NF-κB, and CREB are stimulated by oxidants [127,131,157]; it is possible that estrogen specific effects at the level of mitochondria can activate these transcription factors. Based on these studies we postulate that estrogen-induced mitochondrial ROS stimulates oxidant sensors a-raf, Akt, or PKC, which in turn activate transcription factors such as NF-κB, CREB, or AP-1 via the MEK/ERK pathway resulting in the transcription of cell cycle genes containing DNA responsive elements for NF-κB, CREB, or AP-1 and ultimately estrogen-induced cell proliferation (Fig. 1).

#### Estrogen and mitochondrial-cytoskeleton interactions

Mechanical signals associated with cytoskeletal tension generation and cytoskeleton restructuring are a requirement for anchorage dependent cells to pass through the late G_1 _restriction point [158]. Since these cytoskeleton dependent effects on the G_1 _checkpoint are independent from the MAPK signaling pathway, a new question rises of whether mitochondria can modulate cell growth by interacting with the cytoskeleton. Mitochondria are reported to be associated with three major cytoskeletal structures, which include microtubules, microfilaments of actin, and intermediate filaments [159]. Mitochondrial tubulin and microtubule associated proteins (MAPS) are reported to bind to porin or VDAC a component of the permeability transition pore [160]. The association of the cytoskeleton with VDAC could be biologically significant because the actin filament severing and capping protein gelsolin has been reported to modulate ΔΨ_m _by its interactions with VDAC [161].

Epithelial cell spreading results from the binding of integrins to the extracellular matrix which depends on the actin cytoskeleton [162,163]. The survival of epithelial cells depends on this interaction with the extracellular matrix, which if disrupted leads to a specific form of apoptosis called anoikis [164]. Actin filaments are necessary to cluster integrin receptors and proteins linked to their cytoplasmic domain into focal adhesion complexes [165]. These focal adhesion complexes provide a direct link between the extracellular matrix and the actin cytoskeleton. Anchorage-independent growth is a property of cancer cells, which may depend on the mitochondria based on evidence from the following studies. Long-term exposure of cells to ethidium bromide, an intercalation agent which inhibits mtDNA replication, results in the depletion of mitochondria. Mitochondria-depleted (ρ°) brain and breast tumor cells have been shown to lose their ability for anchorage-independent growth [166]. In addition, human ρ° cell lines derived from ovarian carcinoma, cervical carcinoma, and osteogenic sarcoma were demonstrated to be non-tumorigenic or poorly tumorigenic when administered subcutaneously to nude mice [167]. Taken from these reports is the interesting possibility that cancer cells maintain tension on the cytoskeleton via the contraction and expansion of mitochondria instead of binding to the extracellular matrix. Actin assembly and disassembly is regulated by the protein gelsolin. Since gelsolin is reported to prevent apoptotic mitochondrial changes by binding and closing VDAC, perhaps other diverse functions are modulated by interactions between the mitochondria and cytoskeleton. More recently, it was demonstrated that mitochondrial ROS production is stimulated by integrin induced changes [73]. Although integrin receptors are linked to the actin cytoskeleton, it is not clear whether the signal that is transduced to the mitochondria occurs via the cytoskeleton. Furthermore microtubules have also been implicated in the biogenesis of mitochondria based on the inhibition of mitochondrial mass increase and mtDNA replication caused by the microtubule-destabilizing drug colchicine and the estrogen metabolite 2-methoxyestradiol in mammalian cells [168].

Mitochondria biogenesis is reported to occur in the G_1 _phase of the cell cycle but also starts in the late S phase [169]. Although mitochondria were not reported to be an upstream signaling event for generating cytoskeleton tension, physical effects of mitochondria such as shape changes and stretching or contraction could generate tension based on its association with the cytoskeleton. Changes in cytoskeleton tension may be mediated by mitochondria in response to estrogen. For instance, transmission electron microscopy (TEM) showed an increase in mitochondria size of MCF7 cells treated with E2 [14]. This change in mitochondrial size may generate mechanical forces that, in turn, may transduce a signal to the nucleus. Another possibility is that estrogen stimulated mitochondrial ROS may affect the elasticity of the actin network. Actin filaments are a prevalent feature of the cytoskeleton, which partially determine the overall mechanical strength of a cell. Thiol oxidation forms disulfide-bonded actin dimers resulting in interfilament cross-links [170]. Estrogen-induced oxidative stress has been recently reported to oxidatively modify cysteine residues of proteins [137]. Thus, estrogen-induced mitochondrial ROS could stimulate the formation of actin dimers that modulate cytoskeleton tension which in turn transduces a signal to the nucleus (Fig. 1).

#### Mitochondrial proteolysis and peptides as signals

The mitochondrial protein cytochrome c is known to be released to the cytosol where it initiates a signal for apoptosis. Given the role of cytochrome c the existence of other mitochondrial protein signaling molecules is a likely possibility that could mediate a diverse number of cellular processes including cell growth. It has been shown that mitochondria have the capability to export mitochondrial-matrix proteins to other cellular compartments such as the nucleus, peroxisome, endoplasmic reticulum, and secretory vesicles [171]. For example, mitochondrially transmitted factors (MTFs) are peptides derived from mitochondrial encoded proteins that are presented on the cell surface as minor histocompatability antigens. MTFs are derived from the mitochondrial encoded NADH dehydrogenase subunit 1 gene in murine and humans while rat MTFs are derived from the mitochondrial encoded ATPase 6 gene [172,173]. Although the synthesis and cell surface expression of MTFs was inhibited by the mitochondria specific protein synthesis inhibitor chloramphenicol, it is not clear whether post-translational modifications of mitochondria proteins are also responsible for MTFs; given that chloramphenicol is also an inhibitor of proteolysis in rat liver mitochondria [174]. Thus, mitochondria may serve as a subcellular compartment of proteolysis that generates signaling peptides that are exported to the cytosol. It is possible that proteolysis plays a significant role in mitochondrial protein processing because the chemical rhodamine 6G (R6G) inhibits matrix catalyzed processing of rat-liver mitochondrial precursors which include iron-sulfur protein, cytochrome c_1_, and core protein I of the cytochrome bc_1 _complex; the α and β subunits of F1 ATPase and subunit IV of cytorochrome oxidase [175]. The molecular mechanism of R6G inhibition of protein processing was not identified, but it was proposed to be due to an interaction between R6G and a matrix protease. The import of proteins into mitochondria has been investigated in great detail while the process of export is minimally explored. A novel ATP-binding cassette (ABC) transporter, Mdl1, located in the inner mitochondrial membrane of yeast is required for the export of mitochondrial peptides with a molecular mass of 2100 to 600 daltons generated by proteolysis [176]. It was suggested that the export of peptides from the mitochondria may allow the mitochondria to communicate with its environment. This novel mode of communication may exist based on studies that demonstrate mitochondrial specific cleavage and export of cytokines. The cytokine IL-1β is localized in the mitochondria of LPS stimulated human peripheral blood monocytes and the 31 kDa form of IL-1β is reported to be cleaved into the 17 kDa mature active form within the mitochondria upon exposure to the HIV coat glycoprotein 120 [177,178]. Since macrophages secrete IL-1β by the ABC transporter, it is possible that proteins up to 17 kDa may be exported from the mitochondria [179]. In addition to IL-1β, mitochondrial Trx protein may also participate in a similar protein export mechanism. A truncated form of Trx, Trx80, is reported to be a potent mitogenic cytokine, however, it is not known whether Trx80 is derived from mitochondrial Trx [180]. Based on these reports it appears that cleaved proteins may be released as growth factors in response to an estrogen-induced rise in mitochondrial calcium that activates proteolysis. Thus, cleaved signaling peptides from the mitochondria may stimulate cell growth in an autocrine manner (Fig. 1).

Several proteases are known to exist in the mitochondrial matrix such as ATP-dependent human Lon protease, ATP-dependent human Clp proteinase chain P (hClpP), and Ca^2+ ^dependent neutral protease [181-183]. Since endogenous proteolysis is a mechanism that regulates cell cycle progression, we postulate that E2-induced rise of mitochondrial calcium can activate calcium dependent proteases such as calpeptin and possibly hClpP. Once activated mitochondrial matrix proteins could be cleaved into bioactive forms that are exported to the cytosol. The heterogeneous association between ERα cleavage products and regulatory proteins has been suggested to play a role in physiological or pathological processes [184]. Low molecular weight ERα isoforms (~35–28 kDa) have been identified in mitochondria [16]. The 44-kDa protein related to the nuclear RXRα receptor is reported to be enzymatically cleaved and imported into the mitochondrial matrix [185]. It may interact with mitochondrial proteins or bind the organelle genome. Once in the cytosol mitochondrial clevage products may also regulate the function of various enzymes. For example, a truncated ERα 46-kDa protein in human endothelial cells mediated an acute activation of eNOS in response to a 15 min E2 (30 nM) treatment [186]. A significant finding from this report is that the truncated ERα 46-kDa mediates acute responses of estrogen rather than transcriptional responses in endothelial cells. Recently, it was reported that physiologic concentrations of E2 (<10 nM) induced NOS1 and activates the cGMP signal transduction pathway leading to sustained expression of Trx and MnSOD in human SH-SY5Y cells [187]. Thus, the acute activation of mtNOS by ERα cleavage products is yet another interesting possibility for redox signaling.

## Conclusion

In summary, mitochondria are a major target of estrogen. It appears that mitochondria through its interaction with the cytoskeleton, export of cleaved signaling peptides, and/or generation of ROS may transduce signals to the nucleus for the activation of transcription factors, such as, AP-1, NF-κB, and CREB involved in the cell cycle progression of estrogen-dependent cells. These interactions between estrogen and mitochondria merit furture investigations, which may shed new light on the role of mitochondria in cell growth.
